# *Dichrostachys cinerea *(L.) Wight *et *Arn (Mimosaceae) hydro-alcoholic extract action on the contractility of tracheal smooth muscle isolated from guinea-pig

**DOI:** 10.1186/1472-6882-11-23

**Published:** 2011-03-17

**Authors:** Raissa RR Aworet-Samseny, Alain Souza, Fidele Kpahé, Kiessoun Konaté, Jacques Y Datté

**Affiliations:** 1 Institut de Pharmacopée et de Médecine Traditionnelle, Centre National de la Recherche Scientifique et Technologique (Cenarest) BP: 1156 Route de Sibang 3 Libreville Gabon; 2 Laboratoire de Nutrition et de Pharmacologie, UFR BioSciences, Université de Cocody, Abidjan 22 BP: 582 Abidjan -Côte d'Ivoire; 3 Laboratoire de Biochimie et de chimie Appliquées, UFR Science de la Vie et de la Terre, Université de Ouagadougou, 09 BP 848 Ouagadougou 09 Burkina Faso

## Abstract

**Background:**

*Dichrostachys cinerea *(L.) Wight et Arn. (Mimosaceae) is largely used in ethno-medically across Africa, and mainly employed for the treatment of asthma in Ivory Coast and Gabon. The paper analyses the relaxation induced by the methanolic extract of *D. cinerea *(Edici) in the guinea-pig trachea preparations (GPTPs). Purpose: This study aimed to bring out the scientific basis to the use of this plant leading to the validation of this phytomedicine.

**Method:**

The aorta obtained from guinea-pigs was immediately placed in a Mac Ewen solution. Experiments were performed in preparations suspended between two L-shaped stainless steel hooks in a 10 ml organ bath containing Mac Ewen solution. The isometric contractile force of the aorta strips of guinea-pig were recorded by using a strain gauge. The different drugs were directly administered into the organ bath and the magnitude of GPTPs was evaluated.

**Results:**

Phytochemical analysis of the methanolic extract of Dichrostachys *cinerea *(Edici) using chemical methods revealed the presence of flavenoids, tannins, sterols, triterpenes and polyphenols. Pharmacological studies performed in GPTPs show that of *Dichrostachys cinerea *(0.1 mg/ml - 2 mg/ml) evoked a broncho-constriction in GPTPs. Whereas, at concentration up to 2 mg/ml, Edici induced a significant dose-dependent relaxation in the GPTPs. KCl-, ACh- or histamine-evoked contractions of isolated trachea was significantly inhibited by increasing concentrations of Edici (3.5-10 mg/ml). Edici (10 mg/ml) as well as promethazine (0.25 mg/ml) significantly inhibited contractions induced by increasing concentrations of histamine (1×10^-7^-1×10^-4^mg/ml). In the presence of atropine at a concentration of 10^-6^mg/ml, contractile response curve (CRC) evoked by ACh (1×10^-5^-1×10^-2 ^mg/ml) was significantly abolished in concentration-dependent manner. Edici did not significantly reduced ACh evoked contraction (10^-5^-10^-2^mg/ml).

**Conclusion:**

These observations suggest that Edici could act through two mechanisms: firstly by activation of β-adrenergic or histaminergic receptors; and secondly muscarinic receptors may not be greatly involved, that justifying the use of the extract in traditional Medicine in Africa.

## Background

Asthma is a chronic inflammatory disorder of the airways characterized by airways obstruction, airways hyper-responsiveness, excessive mucous secretion and cough [1]. Nowadays, asthma represents a public health problem in African countries with a prevalence ranging from 1 to 12% according to WHO estimation.

Although asthma cannot be totally cured, appropriate management can control the disease and enable people to enjoy a good quality of life. Short-term medications are used to relieve symptoms. People with persistent symptoms have to take long-term medication daily to control the underlying inflammation and to prevent symptoms and exacerbations.

Folk medicine provides many phytomedicines which represent a significant alternative for the management of this affection in several communities around the world. In India, *Solanum xanthocarpum *is used for the management of bronchial asthma [2]; in Europe *Inula helenium*, has been used since the middle ages for its expectorant properties, it is known as a stimulant to the respiratory system and has long been used to treat asthma and chronic bronchitis [3]. In Ivory Coast traditional medicine, air-dried powdered stem bark of *Dichrostachys cinerea *is used by inhalation for the treatment of this airways affection [4,5].

*Dichrostachys cinerea *which belongs to the family of mimosaceae is also used for the treatment of wounds, rheumatism and renal troubles [6]. Pharmacological report on D. *cinerea *has shown antibacterial effect [7,8] and antiviral. Several authors have shown that the species inhibit protein farnesyl-transferase activity [9,10]. Moreover, chemical studies revealed the presence of a new isomer of mesquitol (a main active principle), which shown free-radical scavenging property and α-glucosidase inhibitory activities [11].

Phytochimical studies performed on *D. cinerea *extracts have revealed the presence of tannins, sterols and triterpenes, of reductionist compounds, polyphenols, flavenoids as well as of cardiotonic heterosides [12].

Based on the fact that guinea-pig airways display many anatomical, physiological and pharmacological attributes of human airways [13] and that the smooth muscle of trachea represent an ideal model for the study of the airway regulation [14], the hydro-alcoholic extract of *D. cinerea *was performed on the isolated trachea rings of the guinea-pig in order to evaluate its therapeutic potential for the management of asthma. Our work aims to bring out scientific support to the use of *Dichrostachys cinerea *as antiasthmatic remedy in folk medicine. Since tracheal muscle cells of guinea-pig have similar physiological properties than human ones [15,16], the present investigation is a comparative study of *Dichrostachys cinerea *methanolic extract to histamine in isolated guinea-pig's tracheal smooth muscle.

## Methods

### Plant material

*Dichrostachys cinerea *(L.) Wight et Arn. (Mimosaceae) is a shrub up to eight feet high, with branches ending in thorns. The leaves are bipinnate, each pinna bearing a gland. The pendant flowers 2.5 cm long are composed of an upper and a yellow hermaphrodite bottom sterile from purple to pink. The fruit pods are twisted, indehiscent, a decorative effect original. It also comes growing on heavy soils are locally abundant and characteristic of savannas. This species occurs in Central, Southern and tropical Africa.

Fresh roots barks of *Dichrostachys cinerea *were collected at Essassa (December 2009, rain season) in the province of Estuaire (Gabon). The plants were authenticated by Mr Raoul Niangadouma a botanist of Gabon National Herbarium (IPHAMETRA/CENAREST). A voucher specimen (H.P Bouroubou 387, M.S.M Sosef n°: 894, M.SM 1097) were deposed in this department.

### Preparation of the plant extract

The sun-dried roots barks of *Dichrostachys cinerea *were cut into small pieces, using a micro-crusher (Retsch SK 100 Confort Geissen Germany). The fine powder obtained was firstly macerated (100 g) for 24 hours in petrol ether (500 ml) using magnetic stirrer to remove oils, chlorophylls. Solvent was removed and the solid remainder was collected and dried at room temperature. It was macerated again in methanol (500 ml) for 24 hours. The supernatant was filtered with Wattman paper and it was evaporated using Rotavapor (Laborota 4002-Control Heidolph, Germany). The methanolic extract of the plant (1.53 g) was obtained and stored at 4°C until experiments.

### Phytochemical screening

Phytochemical study of the methanolic extract was performed using described classical procedures [17,18]. Chemical groups characterized were alkaloids, tannins, flavonoids, polyphenols, saponins, sterols and triterpenes, glycosides, sugars.

### Animals

Guinea-pigs (*Cavia porcellus*) weighing 300-500 g, were used for pharmacological studies. Animals were raised at room temperature (27 ± 2°C) with a natural light-dark cycles with food and water *ad-libitum*. Animals were cared for and treated according to the principles for the care and use of laboratory animals for biomedical research approved by the ethical committee of Cocody University, Abidjan.

### Preparation of isolated tracheal strips

The methods were previously described [19,20]. The animals were sacrificed by a blow on the head and were ex-sanguinated. Trachea was quickly dissected and cleaned of connective tissues. The isolated trachea was cut transversally into small rings which were transferred in a Petri dish containing a Mac Ewen solution (mM) with following composition: NaCl, 130; KCl, 5.6; CaCl_2_, 2.6; NaH_2_PO_4_, 0.91; NaCO_3_H, 11.9; MgCl_2_, 0.24; glucose, 11. The preparations were transferred in normal Mac Ewen solution.

### Measurement of isometric tension

Tracheal isolated preparations were mounted in 10 ml jacketed tissue baths by suspending them between two L-shaped stainless steel hooks. The lower hooks were attached to stationary support such that they could be positioned at the bottom of the bath chambers. The upper hooks were attached to force-displacement transducers (F30, type 372). The bath chamber contained the appropriate Mac Ewen solution (37 ± 0.5°C) constantly bubbled with 95% O_2 _-5% CO_2_, giving a pH of 7.4. Isometric force was measured and recorded using a Multipen Recorder Rikadenki polygraph (Hugo Sachs Instruments, Freiburg, Germany). A pre-load of 1 g was applied. After mounting, strips were equilibrated for 30 min. Changes in isometric force were measured and recorded by means of a force transducer.

### Experimental Protocol

The effects of Edici were evaluated on contractile activity of isolated tracheal strips. The same protocol was used [21]. The rings were mounted as already described [22]. After the 30 min period of equilibration, the contractile response to depolarizing potassium solution was assessed as a test for viability. The depolarizing KCl solution (80 mM) had the same composition as the Mac Ewen solution used, except for the NaCl that had been completely replaced by an equimolar amount of KCl. The GPTPs were washed with Mac Ewen solution four times and re-equilibrated for another 30 min. Edici was directly administered for 4 min in the organ bath and the magnitude of the contractile activity of the GPTPs was evaluated. After equilibration for 30 min, cconcentration-response curve (CRC) for plant extract, histamine or ACh were obtained by cumulatively added increasing concentrations.

Edici was directly administrated into the organ bath and the contractile force was evaluated. In our experiments, the cumulative CRC for either Edici or salbutamol was recorded. In a second step, CRCs for Edici was obtained in GPTPs pre-contracted either with KCl, histamine or Acetylcholine (ACh). To know if Edici acts via histaminergic receptors stimulation, CRC for histamine was realized in GPTPs pre-treated for 15 min with either Edici employed at a unique concentration of 10 mg/ml or promethazine (0.25 mg/ml). In order to study the involvement of cholinergic receptors in the plant extract-induced the trachea relaxation, CRC acetylcholine was realized cumulatively in GPTPs rings pre-incubated with either Edici or atropine (1×10^-5^mg/ml), cholinoceptors antagonist.

Cumulative CRC were analysed using GraphPad software (GrapPad Software Inc., San Diego, CA, USA) to determine pEC_50 _values [negative logarithm of the concentration eliciting 50% of the maximal contractile response (E-max)]. When a plateau in the concentration response curve was not reached, the response observed with the highest concentration of Edici used (10 mg/ml) was considered as E-max.

### Drugs

Acetylcholine, histamine and atropine were purchased from Sigma chemical (St. Louis Mo). Promethazine were obtained from Prolabo (France). Salbutamol [23] was purchased from GlaxoSmithKline (Parma, Italy). All drugs were dissolved in distilled water for the preparation of stock solution.

### Statistical analysis

All values in the text and illustrations are presented as mean ± SEM, with *n *representing the number of different preparations. Differences between pEC_50 _and E-max values of the compounds were evaluated with Dunnett's *t*-test, once an analysis of variance (ANOVA) for data had revealed that the samples represented different populations. Values of *p *< 0.05 were considered to indicate significant differences.

## Results

### Phytochemical screening

Phytochemical study of *Dichrostachys cinerea *shows that the methanolic extract is rich in phenol compounds (phenols compounds and flavenoids). Tannins, sterols and triterpenes were also found in this extract.

### Effect of extract of *Dichrostachys cinerea *on isolated guinea pig tracheal preparations

As shown in table 1, the hydro-alcoholic extract of *Dichrostachys cinerea *(Edici) exerted a biphasic action on a tracheal smooth muscle isolated from the guinea-pig. Edici at concentrations ranging from 0.1 mg/ml to 2 mg/ml induced the GPTPs contractions from 134 ± 32 mg (control value) to 353 ± 49 mg (p < 0.05). However, at concentrations up to 2 mg/ml, Edici evoked significantly a dose-dependent vasorelaxation of the GPTPs smooth muscle from -134 ± 49 mg to -276 ± 51 mg (p < 0.05) at concentrations of 3.5 mg/ml and 10 mg/ml, respectively (Table 1).

**Table 1:** Concentrations of *Dichrostachys cinerea *(Edici) ranging from 0.1 mg/ml to 10 mg/ml on contractile force of strips isolated from guinea-pigs tracheal smooth muscle

C [mg/ml]	0.1	1	2	3.5	10
CF [mg]	+134 ± 32	+215 ± 61	+353 ± 49	-134 ± 49	-276,33 ± 51

C = concentration; CF = Contractile force,

Values are mean ± SEM, n = 4

Similar results were obtained with the pharmacological reference drug, salbutamol, a short-acting β_2_-adrenergic receptor agonist used for the relief of bronchospasm in conditions such as asthma and chronic obstructive pulmonary [30]. The table 2 shows the effect of cumulative doses of salbutamol on the GPTPs. Salbutamol at concentrations of 2.3×10^-6 ^mg/ml to 2.3×10^-5 ^mg/ml induced a contraction of the GPTPs from 109 ± 15 to 183 ± 9.9 mg (p < 0.01). At a concentration up to 2.3×10^-5 ^mg/ml, salbutamol caused relaxation of the GPTPs. The both concentrations of salbutamol (2.3×10^-4 ^mg/ml and 2.3×10^-3 ^mg/ml) caused a significant relaxation of the GPTPs smooth muscle of -85 ± 7.3 mg and -670 ± 20 mg (p < 0.01), respectively (Table 2).

**Table 2:** Concentrations of salbutamol ranging from 2.3×10^-6^mg/ml to 2.3×10^-3 ^mg/ml on contractile force of strips isolated from guinea-pigs tracheal smooth muscle

C [mg/ml]	2.3×10^-6^	2.3×10^-5^	2.3×10^-4^	2.3×10^-3^
CF [mg]	+109 ± 15	+183 ± 9.9	- 85 ± 7.3	-670 ± 20

C = concentration; CF = Contractile force,

Values are mean ± SEM, n = 4

**Figure 1:**
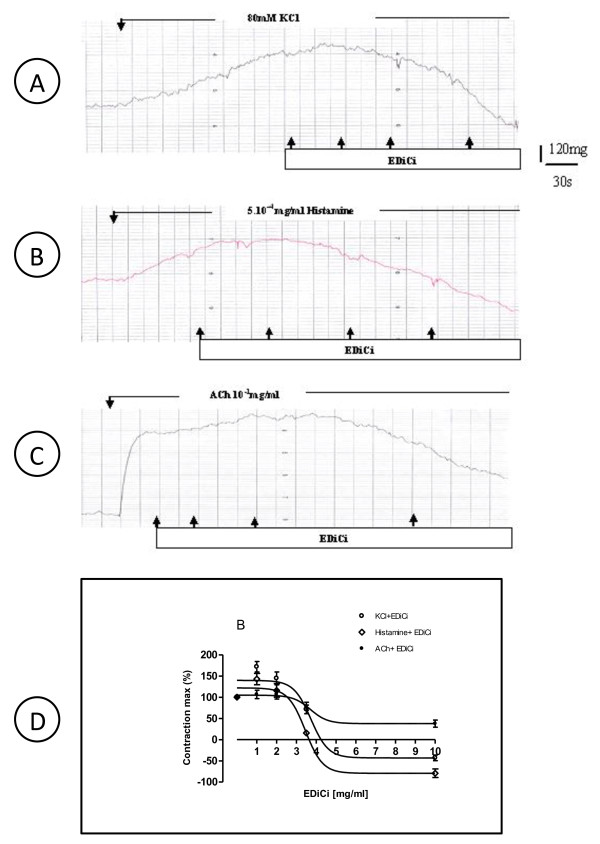
**Original recording of contractile force of the activity of a strip isolated from guinea-pigs tracheal smooth muscle in Mac Ewen solution and the effect of KCl (1A); of histamine (5-HT) (1B) and ACh (1C) in the presence of of *Dichrostachys cinerea *extract (Edici) in concentrations ranging from 1 mg/ml; 2 mg/ml to 10 mg/ml**. The horizontal bar shows the time of recording and the vertical bar represents the magnitude of contractions. Concentration-response curves for *Dichrostachys cinerea *extract (Edici) in isolated tracheal preparations of guinea-pigs in the presence (O) of KCl (80 mM), of (◊) Histamine (5×10^-4 ^mg/ml) and (●) ACh at a concentration of 1×10^-1 ^mg/ml (Figure **1D**)

Original tracings of the guinea pig tracheal activity in the presence of Edici on histamine (H), KCl- or ACh-induced contractions were recorded as shown on Figures 1A, B and 1C, respectively. These effects were graphical represented in figure 1D. The maximum responses to KCl (80 mM), histamine (5×10^-4 ^mg/ml) or to ACh (1×10^-1 ^mg/ml) were significantly reduced by Edici (1×10^-6 ^mg/ml). Indeed, respective E-maximums responses induced by KCl, histamine or by ACh were significantly blocked from 256 ± 16 mg (control value) to -110 ± 7.7 mg for KCl (p < 0.01; EC_50_-value = 0.54×10^-4 ^mg/ml), from 231 ± 14 mg/ml to -183 ± 11.1 mg/ml for 5-HT (P < 0.01; EC_50_-value = 0.28×10^-4 ^mg/ml), induced contracts and from 1268 ± 30 mg/ml to 48 ± 9.1 mg for ACh (p < 0.01; EC_50_-value = 0.50×10^-4 ^mg/ml).

### Comparative study of extract of *Dichrostachys cinerea *and promethazine on histamine-induced contractions on isolated guinea pig trachea strips

**Figure 2:**
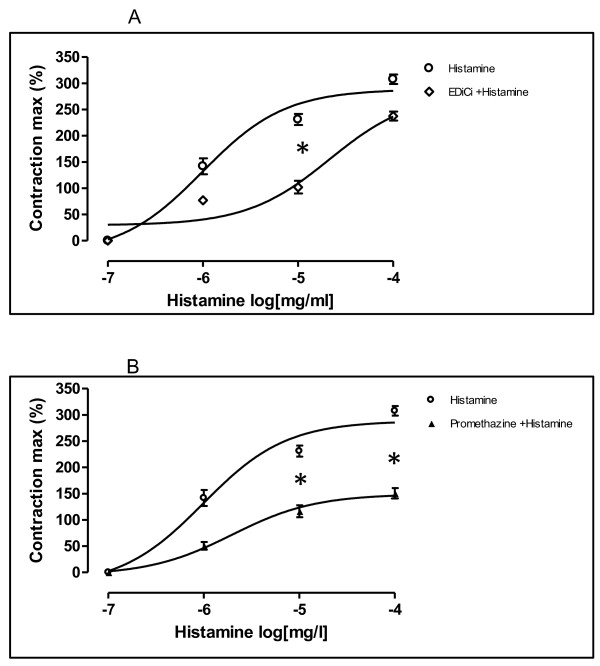
**Concentration-response curves for histamine in isolated tracheal preparations of guinea-pigs in the absence (O) and presence (◊) of Edici (10 mg/ml) (2A), and in the presence (▲) of promethazine at a concentration of 0.25 mg/ml (2B)**. Concentration of histamine are expressed as the log10 of the molar concentration. Each Point represents the mean values obtained using 6 rings. Each obtained from different animal. Brackets indicate SEM (n = 4-6).

Histamine (1×10^-7^-1×10^-4^mg/ml) induced a concentration-dependent increase of the GPTPs. The maximum value (E-max) recorded was 420 ± 60 mg (EC_50_: 9.8×10^-7 ^m/ml). As shown in figure 2A, in the presence of Edici (10 mg/ml), the contractions of the GPTPs induced by histamine were significantly reduced in a concentration-dependent fashion (p < 0.05) compared to maximum response of control group. The E-max in these conditions was 162 ± 16.33 mg with an EC_50_-value of 2.1× 10^-7 ^mg/ml. Similarly, results were obtained with promethazine at a single concentration of 0.25 mg/ml. Contractions elicited by histamine was significantly blocked in the presence of promethazine, a H_1_-histaminergic receptor antagonist (p < 0.05) (Figure 2B). The E-max of the GPTPs tension of histamine was drastically reduced to 183 ± 9.9 mg (EC_50_: 1.9×10^-6 ^mg/ml).

### Comparative effects of extract of *Dichrostachys cinerea *and atropine in ACh-induced contractions of the guinea pig trachea preparations

**Figure 3:**
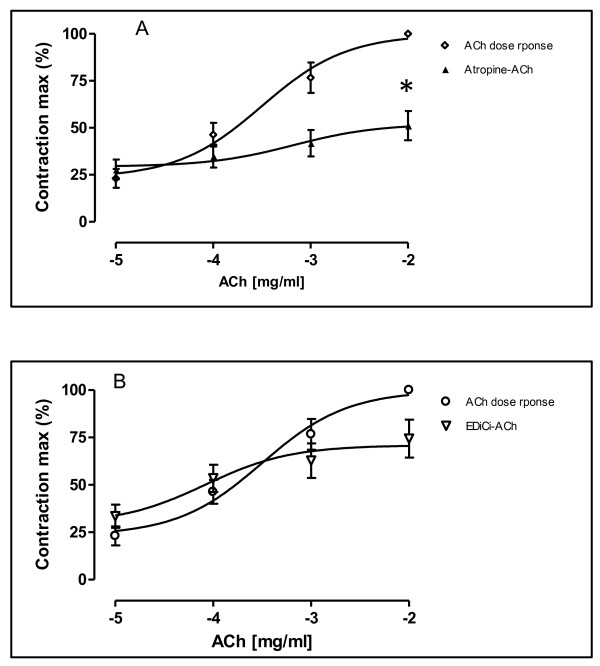
**Concentration-response curves for ACh in isolated tracheal preparations of guinea-pigs in the absence (◊) and in the presence (▲) of atropine (0.25 mg/ml; 3A), and pre-treatment (Δ) with Edici at a single concentration of 10 mg/ml (3A)**. Concentration of ACh are expressed as the log10 of the molar concentration. Each Point represents the mean values obtained using 6 rings. Each obtained from different animal. Brackets indicate SEM (n = 4-6).

Acetylcholine (ACh) in concentration ranging from 1×10^-5 ^mg/ml to 1×10^-2^mg/ml, added cumulatively on the organ bath, induced a significant increase of contractions of the GPTPs in a concentration-dependent manner. The EC_50_-value was 6.62×10^-4^mg/ml. The Maximal value of contraction (E-max) of the concentration-response curve for ACh in of the GPTPs in the presence of atropine (1×10^-5^mg/ml) was reduced to 51.14%. ACh-induced contractions were significantly inhibited. The EC_50_-value was 6.62×10^-4 ^mg/ml (Figure 3A). Similar results were obtained in the pre-treatment of the GPTPs with EdiCi at a unique concentration of 10 mg/ml. The E-max of the concentration-response curve for ACh shifted rightwards significantly. A reduction of E-max-value was 25.57% compared to control value. Moreover, the EC_50_-value was 3.2×10^-4 ^mg/ml in the absence of Edici, and 8.3× 10^-5 ^mg/ml in the presence of EdiCi (Figure 3B).

## Discussion

The powder of sun-dried root barks of *Dichrostachys cinerea *is usually used in folk medicine for the treatment of asthma. The bark powder of *Dichrostachys cinerea*'s root is used in nasal instillation.

In the present work, phytochemical investigation performed on the plant species collected in Libreville (Gabon) forest revealed the presence of bioactive compounds such as alkaloids, flavenoids, tannins, sterols and triterpenes, phenolic compounds. These results are in accordance with those showing the presence of terpenoids, sterols and tannins in the leaves and fruits. Pharmacological study showed a transient bronchoconstriction followed by a sustained bronchodilation in the airway smooth muscle. Similar results were demonstrated with *Parquetina nigrescens *(Periplocaceae) on smooth muscle preparations [24]. These results suggest that constituents in the extract of *D. cinerea *may exert airways regulation.

It is well established that the salbutamol (β2-adrenergic receptor agonist) activity is due to the stimulation β-adrenergic receptors [25]. The fact that *D. cinerea*-evoked biphasic action and mainly bronchodilatory effect was similar to that induced by salbutamol, allowed us to suggest that *D. cinerea*'s active principles may act via β-adrenergic receptors to elicit the relaxation of the trachea smooth muscle. In the GPTPs, salbutamol produces more relaxant effect than salmeterol, suggesting that salmeterol is a partial beta-2 agonist. Very high concentrations of salmeterol as well of salbutamol may induce non-beta adrenoceptor mediated relaxation. There is no pronounced difference in the magnitude of antagonism against carbachol induced contractions between salmeterol and salbutamol [26]. Since, the plant extract exhibited a direct relaxation of normal smooth muscles of the trachea [27] and in addition, it induced a concentration-dependent relaxation of the GPTPs pre-contracted with KCl or with histamine. However, *D. cinerea *hydro-alcoholic extract moderately reduced the bronchoconstriction evoked by acetylcholine. This result did not suggest a high inhibitory effect the plant extract actives principles on the cholinergic receptors. Since, ACh still induced a significant bronchoconstriction on the isolated trachea pre-treated with the plant extract; while atropine, an antagonist of cholinergic muscarinic receptors totally abolished the ACh-evoked at the same conditions. Indeed, the agonist of cholinergic receptors such as ACh evoked bronchospasm which is inhibited by anticholinergic drugs such as atropine [28]. Anti-cholinergic drugs block muscarinic effect of ACh on the receptors of postjunctional membranes and so inhibit the answer of the post ganglionic parasympathetic nerve. The loss of M_2 _muscarinic receptor function occurs in asthmatics and it contributes to bronchial hyper -responsiveness and it is not a chronic feature of asthma, and instead it characterizes asthma exacerbation. The loss of M_2 _muscarinic receptor function in children and adults happens during antigen bronchoprovocation or during exposition of asthmatics to ozone. The research findings support the hypothesis that beta2-adrenoceptor agonist drugs, administered over time *in vivo *or *in vitro*, induce a transient hyperresponsiveness of airway smooth muscle to cholinergic bronchoconstrictor stimuli [29,30]. Histamine-evoked broncho-constriction is due to the activation of H_1_-histaminergic receptors and is blocked by antihistaminergic drugs such as promethazine [31,32]. Whereas, KCl-induced broncho-constriction results from the depolarization of cell membrane which causes the augmentation of calcium influx through voltage operated calcium channels leading to the rise of intracellular calcium level [33]. Moreover, the acute and relatively refractory hyperkalemia can develop. In such situations, incorporating salbutamol with a conventional anti-hyperkalemia strategy can provide an effective therapeutic option to treat hyperkalemia, even during the anhepatic stage [34,35]. Our study showed that, the plant extract, totally inhibited the KCl-evoked contractions and similarly to promethazine, it abolished histamine-evoked contractions suggesting the involvement of different possible mechanisms: a potassium channels opening effect, an inhibitory effect on calcium channels and/or an antagonistic effect on histaminergic receptors. Further studies are required to clarify the contribution of these mechanisms of action.

Pathophysiology of asthma is characterized by two phases, namely a bronchoconstriction followed by a special type inflammation [36]; the bronchoconstriction being the main component of the immediate phase of asthmatic response on multiple stimuli [37]. Overall, the plant extract's actives principles-evoked relaxation on pre-contracted tracheal chains (pathological tissues) highlighted a bronchospasmolytic action that may support its potential usefulness in the treatment of bronchoconstrictive diseases. Regarding the phytochemical composition of the plant extract, active constituents in the plant extract such as terpenoids may be responsible for the bronchodilator action [38]. In addition, flavonoids and polyphenols contents in plant extract may contribute for the biological activities of the extract and thus the therapeutic potential of *D. cinerea *for the management of asthma. Since, these phytoconstituents have spasmolytic action on smooth muscle [39,40], antioxidant and anti-inflammatory activities [41,42].

Promethazine or Edici associated to histamine induced a relaxation of the GPTPs smooth muscle. The Emax values were at half decrease. It is well known that promethazine is a phenothiazine used as an anti-histamine. Antihistamines such as promethazine compete with histamine for one of the receptors for histamine (the H_1 _receptor) on cells. Promethazine also blocks the action of acetylcholine (anticholinergic effect). The same observation was done with the interaction ACh-Atropine or ACh-Edici. The values of EC_5 _and EC_50 _were very influenced in the presence of our extract. Similar results were obtained with seeds aqueous extract from *Acacia nilotica *[43]. Moreover, experiments with the stem bark of *Mangifera indica *Linn. showed similar results [44]. Our experiments suggest that the aqueous extract of *D. cinerea *could block both the histaminic and muscarinic receptors on guinea-pig trachea.

## Conclusion

From the present study, it could be concluded that *Dichrostachys cinerea *possesses marked bronchorelaxation property complying many facets such as acting via adrenergic receptors activation, potassium channels opening effect, calcium channels blocking and/or by antagonistic action on H_1_-histaminergic receptors justifying its use for the management of asthma in folk medicine. This fact is reinforced by well documented antioxidant and anti-inflammatory properties of its active ingredients. The results corroborate with the traditional use of *D. cinerea *in the treatment of asthma.

## Competing interests

The authors declare that they have no competing interests.

## Authors' contributions

RRRAS, FK and KK carried out the experimental studies and drafted the manuscript. AS and JYD played a role in the writing and editing of the manuscript. AS involved in coordination of study done in Gabon. JYD conceived of the study, participated in the design and coordination of the study, supervised the study and revised the manuscript. All authors read and approved the final manuscript.

## Pre-publication history

The pre-publication history for this paper can be accessed here:

http://www.biomedcentral.com/1472-6882/11/23/prepub
